# Process Development of Sj-p80: A Low-Cost Transmission-Blocking Veterinary Vaccine for Asiatic Schistosomiasis

**DOI:** 10.3389/fimmu.2020.578715

**Published:** 2021-02-23

**Authors:** Adebayo J. Molehin, Sean A. Gray, Cheri Turner, Jennifer Davis, Weidong Zhang, Sabiha Khatoon, Madison Rattan, Rebecca Kernen, Christopher Peterson, Souad R. Sennoune, Darrick Carter, Afzal A. Siddiqui

**Affiliations:** ^1^ Center for Tropical Medicine and Infectious Diseases, School of Medicine, Texas Tech University Health Sciences Center, Lubbock, TX, United States; ^2^ Department of Internal Medicine, School of Medicine, Texas Tech University Health Sciences Center, Lubbock, TX, United States; ^3^ PAI Life Sciences Inc, Seattle, WA, United States

**Keywords:** Sj-p80, calpain, *Schistosoma japonicum*, Asiatic schistosomiasis, veterinary vaccine, water buffalo, Montanide ISA 61 VG

## Abstract

Asiatic schistosomiasis caused by *Schistosoma japonicum* is a neglected tropical disease resulting in significant morbidity to both humans and animals - particularly bovines - in endemic areas. Infection with this parasite leads to less healthy herds, causing problems in communities which rely on bovines for farming, milk and meat production. Additionally, excretion of parasite eggs in feces perpetuates the life cycle and can lead to human infection. We endeavored to develop a minimally purified, inexpensive, and effective vaccine based on the 80 kDa large subunit of the calcium activated neutral protease (calpain) from *S. japonicum* (Sj-p80). Here we describe the production of veterinary vaccine-grade Sj-p80 at four levels of purity and demonstrate in a pilot study that minimally purified antigen provides protection against infection in mice when paired with a low-cost veterinary adjuvant, Montanide™ ISA61 VG. Preliminary data demonstrate that the vaccine is immunogenic with robust antibody titers following immunization, and vaccination resulted in a reduction of parasite eggs being deposited in the liver (23.4–51.4%) and intestines (1.9–55.1%) depending on antigen purity as well as reducing the ability of these eggs to hatch into miracidia by up to 31.6%. We therefore present Sj-p80 as a candidate vaccine antigen for Asiatic schistosomiasis which is now primed for continued development and testing in bovines in endemic areas. A successful bovine vaccine could play a major role in reducing pathogen transmission to humans by interrupting the parasitic life cycle and improving quality of life for people living in endemic countries.

## Introduction

Schistosomiasis remains a major parasitic disease of global health importance. The disease is caused by infection with one of several parasitic trematodes belonging to the genus *Schistosoma*. The three most important species are *S. mansoni, S. japonicum* and *S. haematobium*, each which cause clinically distinct diseases. *S. mansoni* and *S. japonicum* cause hepatic/intestinal schistosomiasis while *S. haematobium* is responsible for urogenital schistosomiasis (1, 2). Schistosomiasis is categorized as a neglected tropical disease occurring in the tropics and subtropics with a conservative estimate of 250 million infected people globally and an additional 779 million people at risk of infection (3–5). Left untreated, schistosomiasis can result in significant morbidity and mortality, with an estimated 1.9 million disability-adjusted life years (DALYs) attributed to the disease (6). Over the last few decades, schistosomiasis control programs have largely been predicated on mass administration of the drug praziquantel (PZQ) with water, sanitation and hygiene (WASH) programs serving as adjuncts. Despite these large-scale control programs, the prevalence and transmission of schistosomiasis have remained largely unchecked while the disease is now gaining foothold in geographical areas previously schistosomiasis-free due to the formation of hybrid parasites from bovine- and human-tropic species (7–10), increasing the urgency to address veterinary schistosomiasis.

*S. japonicum* is a zoonotic trematode causing Asiatic schistosomiasis predominantly in the People’s Republic of China, the Philippines, and Indonesia (4). Asiatic schistosomiasis is thought to be a major health risk to over 50 million people in China with approximately 1 million people and hundreds of thousands of livestock currently infected (11, 12). Mathematical modelling and field studies in Asia that focused on China have shown that bovines - particularly water buffaloes - are the primary source of *S. japonicum* transmission through excretion of eggs in feces (13). A 2007 study involving 238 *S. japonicum*-infected bovines (225 water buffaloes and 13 cattle) revealed that the environmental contamination attributable to these animals was approximately 28.7 million eggs per day (14). Despite considerable efforts to control Asiatic schistosomiasis, the transmission rates and prevalence have been largely unaffected - partly due to the rapidity and frequency of re-infection and the fact that over 40 different mammalian species serve as reservoir hosts for *S. japonicum* (6). Beyond the risk of infection to humans, infection of livestock also imposes additional economic burden through reduced availability of livestock for farming purposes, and reduced size and/or lifespan of diseased livestock (15–17). For sustainable schistosomiasis control, a composite control program with an effective vaccine serving as a fulcrum is urgently required (18, 19).

To date, several *S. japonicum* vaccine candidates have been tried [reviewed in Tebeje et al. (20)] including a 67 kDa *S. japonicum* surface protein (Sj67) (21), the Triose Phosphate Isomerase (SjTPI) (22, 23), a Thyroid hormone receptor beta (SjTHRβ) (24), a *S. japonicum* fatty acid binding protein (SjFABP) (25, 26), a 23-kDa tetraspanin protein (rSjC23DNA) fused to bovine heat shock protein 70 (HSP70) (27), a 26-kDa parenchymal protein (Sj26GST) (28), and the 97-kDa protein Paramyosin (Sj97) (12, 29–31). Several of these, particularly rSJC23DNA, Paramyosin, and SjTPI have shown promise in preliminary protection studies in water buffalo.

Our focus for more than 10 years has been on a human vaccine for *S. mansoni* based on the large (~80 kDa) subunit of calcium activated calpain protein, Sm-p80 (32–36). Sm-p80 has been tested in baboons with multiple adjuvants [reviewed in Zhang et al. (35)] and has demonstrated significant protection from *S. mansoni*-induced liver and intestinal disease. Specifically, this vaccine resulted in reduction of eggs deposited in the liver (91.35%), the small intestine (86.50%), and the large intestine (91.1%). Additionally, the vaccine resulted in a 81.5% reduction in egg hatching efficiency and preferential killing of female worms (91.35%). One embodiment of the vaccine, termed SchistoShield^®^, combines Sm-p80 antigen with the potent TLR4 adjuvant GLA-SE and is set to enter Phase 1 human clinical trials in the US and Phase 1B trials in Africa in early 2021. In addition to protection against *S. mansoni*, we have shown Sm-p80-based vaccination provides partial cross-protection against infections with *S. japonicum* and *S. haematobium* in mice and hamsters/baboons, respectively (37, 38). This evidence of cross-protection provided the rationale to test the p80 orthologs from related *Schistosoma* species to better protect against the homologous pathogen. To this end, we report the cloning, prokaryotic expression, and purification of the full length p80 ortholog from *S. japonicum* (Sj-p80) and pilot efficacy testing in a mouse model of infection and disease. Our preliminary studies suggest that Sj-p80 may emerge as a viable vaccine candidate for Asiatic schistosomiasis, either alone or possibly in combination with other promising candidates, once protection is demonstrated in bovines.

## Materials and Methods

### Animals and Parasites

Female C57BL/6 mice (3–4 weeks old) were purchased from Charles River Laboratories (Wilmington, MA, USA). *S. japonicum* (Philippines strain)-infected *Oncomelania hupensis* snails were procured from the Schistosome Resources Center (Biomedical Research Institute, Rockville, MD, USA).

### Cloning and Expression of Sj-p80

The published sequence of the calpain from *S. japonicum* (Sj-p80) (GenBank #BAA74718.1) was used for cloning (39, 40). The full 2289 nucleotide open reading frame encoding the 758 amino acid Sj-p80 was synthesized and cloned into the expression plasmid pRSET-A by GeneArt Contract Services (Thermo Fisher, Waltham, MA). The Sj-p80 insert was subcloned into the vector pCOLD II (Takara Bio USA, Mountain View, CA) for expression. The resulting plasmid was sequenced bi-directionally for correctness and transformed into the *Escherichia coli* expression strain HMS174. Expression scouting at temperatures ranging from 15°C to 37°C all resulted in the Sj-p80 protein being localized entirely into inclusion bodies (IB).

### Purification of Sj-p80 From Inclusion Bodies

Insoluble IBs were purified by standard protocols following disruption of the bacteria by microfluidization. The IB pellet was washed in 1% CHAPS followed by a second wash in 25% isopropyl alcohol at 10 ml per gram of IB wet weight. The final washed IB pellet was resuspended in 8 M urea/20 mM Tris pH 8.0 (30 ml per gram wet IB weight) and then solubilized overnight at 4°C with gentle rotation. The solubilized IB solution was clarified by centrifugation and Sj-p80 purified as described below.

We subjected the Sj-p80 IB to various purification steps, and samples retained after each resulted in four distinct levels of increasing purity (designated purity 1–4). Purity 1 represents crude IB solution in 8 M urea/20 mM Tris (pH 8.0) without any further purification, buffer exchange, or endotoxin removal. For purity 2, the solution in 8 M urea/20 mM Tris was refolded by dialysis into 3 × 50 volumes of 20 mM Tris (pH 8.0) reducing the urea concentration to < 1 mM. The solution was clarified by centrifugation at 16,000*g* for 60 min followed by filtration to remove residual particulates and concentrated to ~1 mg/ml with 5% glycerol added for protein stabilization. To obtain purity 3, the IB solution in 8 M urea and 20 mM Tris pH 8.0 was negatively passed through the strong cation exchange resin Capto-S (Cytiva, Chicago, IL). This step was included to promote binding of the contaminant proteins while allowing Sj-p80 to flow through with minimal resin binding. The Capto-S flow-through, containing enriched Sj-p80, was again exchanged into Tris, clarified as described above, and concentrated to ~1 mg/ml with 5% glycerol. For purity 4, the Capto-S flow through in 8 M urea and 20 mM Tris (pH 8.0) was bound to the mixed mode chromatography resin Capto-MMC (Cytiva). The column was washed with 10 column volumes of 20 mM NaPO_4_/8M urea/160 mM NaCl (pH 7.0) to remove weakly bound contaminants. Bound Sj-p80 was eluted using a NaCl gradient ranging from 160 mM to 2 M NaCl in 20 mM NaPO_4_/8 M urea (pH 7.0). Eluted Sj-p80 was dialyzed into 20 mM Tris (pH 8.0), clarified, and concentrated as described above.

### Cost Analysis for Production of Vaccine Antigen at Various Purities

We performed calculations to estimate likely production costs to produce and purify the vaccine antigens at all four purities. The 5 production steps included: (1) a 10 L *E. coli* growth and expression, (2) IB isolation and preparation, (3) diafiltration into aqueous (Tris) buffers, (4) a Capto-S chromatographic purification, and (5) a Capto-MMC chromatographic purification; and were itemized to estimate materials costs (buffers, resins, disposables, SDS-PAGE gels, etc.) and personnel costs at a 2020 rate of $170 USD/hr. The costs for each of the 5 steps above were estimated to be $4360 USD for step 1, $1560 USD for step 2, $1520 USD for step 3, and $5040 USD for each of steps 4 and 5. The total cost to produce Sj-p80 at a given purity was derived by adding the costs for each individual step. For example, to obtain Sj-p80 at purity 1 requires steps 1 and 2 for a total cost of $5920 ($4360 + $1560). To obtain cost per milligram, the total cost is divided by the average protein yield. Finally, to obtain the cost per bovine dose, assuming a dose size of 0.25 mg, the cost per mg is divided by 4.

### Detection of Purified Sj-p80 and *Escherichia coli* Host Cell Proteins

For the Sj-p80 immunoblotting, a cross-reactive anti-Sm-p80/Sj-p80 monoclonal antibody (Mab) (SMab4) was selected for use. Proteins were resolved by SDS-PAGE, transferred to PVDF membrane, blocked, and then probed with SMab4 at a concentration of 0.013 µg/ml for 1 h. Bound Mab was detected using goat-anti-mouse IgG (H+L) conjugated to horseradish peroxidase (HRP) (Southern Biotech, Birmingham, AL) at a dilution of 1:2000 and exposed with Ultra-TMB (Promega, Madison, WI). For the anti-*E. coli* HCP Western, blots were probed for 1 h with Rabbit-anti-Ec-HCP polyclonal antisera (Rockland, Limerick, PA) at a dilution of 1:1000 and bound antibody was detected using Donkey-anti-rabbit IgG-HRP (Southern Biotech) at a dilution of 1:2000.

### Immunization Strategy and Experimental Challenge

For the first animal study, 25 mice were divided into five groups (*n*=5/group). Each mouse from the control group received 50 µl phosphate-buffered saline with 50 µl Montanide™ ISA61 VG (SEPPIC, Fairfield, NJ) while mice from the four experimental groups each received a 100-µl injection containing 25 µg Sj-p80 at purities 1 to 4 in a volume of 50 µl mixed with 50 µl of Montanide™ ISA61 VG. Three identical injections each were performed at weeks 0, 4, and 8. *S. japonicum* cercariae were collected from infected *O. hupensis* snails by shedding. Four weeks after the last immunization, each mouse was challenged with 40 *S. japonicum* cercariae by tail immersion method (17). All injections were administered intramuscularly (i.m.). For the second animal study, 30 mice were divided into two groups (*n*=15/group). Each mouse from the control group received 50 µl phosphate buffered saline with 50 µl Montanide™ ISA61 VG (i.m.) while mice from the experimental group each received a 100-µl injection containing 25 µg Sj-p80 at the highest purity (purity 4) in a volume of 50 µl mixed with 50 µl of Montanide™ ISA61 VG.

Mice were given 3 injections and were challenged as described for the first animal study. Mice that were immunized with Sj-p80 purity 1 in 8M urea received 25 µl antigen diluted 1:2 with 25 µl PBS. Prior to the i.m. injection, this was again diluted 1:2 with 50 µl of Montanide™ ISA61VG adjuvant resulting in a final concentration of 2 M urea. This level of urea equals 12.02 mg of urea in a 20 gram mouse which was well below safe limits as previously published for humans (41–44) and mice (45–47).

### Analysis of Total IgG and Antibody Subtypes

Approximately 100 µl of blood was obtained from each mouse prior to all immunizations, cercarial challenge and at euthanasia. Blood was allowed to clot at room temperature and approximately 50 µl serum was obtained after centrifugation. Antibody response following vaccination was determined by Enzyme-Linked Immunosorbent Assay (ELISA) (38). In order to accommodate a total of 6 ELISA assays performed in triplicate measuring total IgG, IgG subtypes, IgA, and IgM, it was necessary to pool the sera from individual mice into groups based on antigen. Briefly, 96-well microtiter plates were coated with 1.2 µg/well of Sj-p80 purity 4 as described previously (38, 48). Diluted sera (two-fold dilutions starting at 1:100) from pooled mice sera (each group) were used as primary antibody and the individual antibody isotypes were detected with HRP-labeled anti-mouse IgG, IgG1, IgG2c, IgG2b, IgA, or IgM (Alpha Diagnostics International, Inc., San Antonio, TX). All assays were carried out in triplicate. Results were expressed as end-point titers determined from the curve of optical density versus serum dilution for the cutoff of two standard deviations above control value (49).

### Worm and Egg Burden Determination

All mice were euthanized six weeks post-cercarial challenge and adult worms recovered from the mesenteric vasculature and hepatic portal system by perfusion. Samples were counted as previously published (17). Percent reduction in adult worm burden was determined by comparing the number of worms retrieved from the experimental groups (I) to the control group (C). Protection (P) was calculated using the formula: %P = [(C − I)/C × 100]. In order to determine the tissue egg load, livers and intestines of the euthanized mice were digested in 4% KOH (17) and the percent reduction in tissue egg retention calculated.

### Tissue Egg Hatching

Tissue egg hatching rates were determined as previously described (33). Briefly, 0.5−1 g of liver and intestine sections were collected from each mouse following euthanasia. Samples were collected and kept in cold 1.2% NaCl on ice throughout the procedure. Tissue samples were finely chopped, suspended in 50 ml PBS (supplemented with 10 µg penicillin and 20 µg streptomycin) and incubated overnight at 37°C with 20 mg of collagenase B (Millipore Sigma, St. Louis, MO, USA). The digested samples were then passed through a series of sieves (425 µm, 180 µm, 106 µm and 40 µm) and *S. japonicum* eggs were collected from the 40-µm sieve followed by centrifugation at 300*g* for 5 min. The supernatant was discarded and egg pellet seeded into 24-well plates and placed under a light source for 2 h to induce egg hatching. Unhatched mature eggs and hatched eggs were counted using a light microscope and egg hatching rates were expressed as the percent of hatched eggs versus overall mature eggs.

### Total RNA Extraction and First Strand cDNA Synthesis

Following animal euthanasia, mouse spleens were collected and splenocytes isolated as previously described (50). In brief, spleens were gently crushed to release splenocytes and washed three times before resuspending cells in complete media (RPMI-1640 supplemented with 10% fetal bovine serum, 100 µg/ml penicillin G, 100 µg/ml streptomycin and 10 µg/ml gentamycin). Spleen cells were seeded at 5 × 10^5^ cells/well into 24-well plates and incubated at 37°C and 5% CO_2_ for 24 h before being stimulated with 1.2 µg Sj-p80 purity 4 and incubated for another 24 h in the same conditions as above. Media alone and Tris buffer were used for control stimulations. Total RNA was extracted using GenElute^®^ Mammalian Total RNA Miniprep kit (Millipore Sigma, St. Louis, MO, USA) according to manufacturer’s instructions. First strand cDNA synthesis was carried out using Maxima First Strand cDNA Synthesis kit (Thermo Fisher Scientific, Waltham, MA, USA) according to manufacturer’s instructions.

### Quantitative Real-Time PCR

Quantitative real-time PCR (qRT-PCR) was carried out to determine the expression levels of a panel of Th1, Th2 and Th17 cytokines. Gene-specific primers were designed from mRNA sequences available from NCBI for *Mus musculus*. Amplifications of target genes were performed using SYBR premix Ex Taq^®^ (TIi RNase H Plus) (Takara, Japan) on a StepOne™ plus Real time PCR platform (Thermo Scientific) in a reaction volume of 20 µl and primer concentration of 0.4 µM. The cycling conditions were initial denaturation at 95°C for 5 min and then amplification for 40 cycles at 95°C for 5 s, 60°C for 30 s. Glyceraldehyde 3-phosphate dehydrogenase (GAPDH) was used as a house-keeping gene. All reactions were carried out in triplicate and results analyzed using DataAssist™ software v3.0. Cytokine mRNA expression profile was determined by comparing the differences in the mRNA levels of the experimental groups with the control group after normalization with GAPDH.

### Statistical Analysis

Statistical analyses were performed using GraphPad Prism version 9. Statistical significance between experimental and control groups was calculated using Student’s unpaired *t*-test. Data were expressed as mean ± SE and a *p* ≤ 0.05 was considered significant.

## Results

### Recombinant Production of Sj-p80

**Figure 1:**
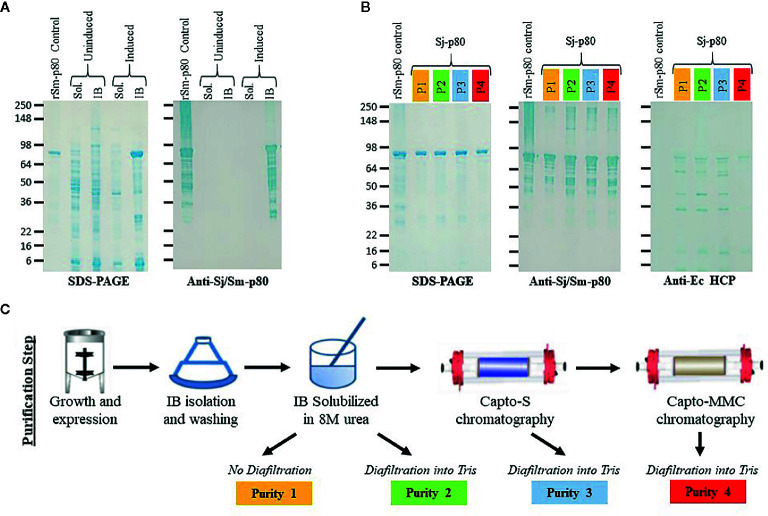
Purification and characterization of Sj-p80. **(A)** Expression of Sj-p80 in the cytoplasmic fraction (soluble) or IB fraction (insoluble) in *E. coli* HMS174 by SDS-PAGE and Western analysis. The primary Sj-p80 band at ~80 kDa is only visible in the induced IB fraction of the SDS-PAGE gel and Western blot following probing with the anti-Sj-p80 monoclonal antibody SMAb4. **(B)** SDS-PAGE (left panel) and Western analysis with SMAb4 (middle panel) comparing the 4 levels of purity of Sj-p80. The rightmost panel shows the abundance of *E. coli* HCPs as determined by Western blotting with anti-*Ec*-HCP polyclonal antibody. **(C)** An overview of the production steps required for purifying Sj-p80 at each distinct purity level.

We successfully expressed Sj-p80 in *E. coli* and found the recombinant protein to be localized almost exclusively to the IB fraction as demonstrated by SDS-PAGE and Western blot (**Figure 1A**). Analysis of crude IBs revealed that they were composed of ~63 +/- 12.1% Sj-p80, and that minimal purification was likely to be needed. Sj-p80 was purified at four levels of increasing purity designated purity 1 (least pure) to purity 4 (most pure). As shown in **Figure 1B**, the Sj-p80 purity was essentially unchanged going from purity 1 to purity 2 with the primary benefit of this step being to refold the protein out of 8M urea and into tris buffer. To obtain purity 3, the Sj-p80 was negatively passed across the ion exchange resin Capto-S. This step was introduced mostly to reduce endotoxins but also resulted in a modest depletion of *E. coli*-derived host cell proteins (HCP). Again, Sj-p80 at purity 3 had purity comparable to protein at purities 1 or 2 with total protein purity (main band at ~80 kDa plus aggregation and degradation band) ranging between 95% and 99% and the purity of the major band at 80 kDa ranging from 60% to 65% (**Table 1**).

**Table 1:** Vaccine cost analysis at 10 L scale.

Parameter	Purity Level (1= lowest purity → 4 = highest purity)			
	Purity 1	Purity 2	Purity 3	Purity 4
Cost/10L production run	$5920	$7480	$12520	$17560
Yield per 10L (mg)	508	486	448	224
Production cost/mg	$11.65	$15.39	$29.94	$78.39
Purity of ~80 kDa	63.9%	65.3%	60.7%	87.8%
Total purity of Sj-p80	97.3%	95.5%	>99%	>99%
Endotoxin level (EU/mg)	*Not tested*	4495	4031	810
Cost per cattle dose (0.25mg)	$2.91	$3.85	$7.49	$19.60

The final purification step involved binding the Sj-p80 on the mixed mode resin Capto-MMC. Following elution, from the Capto-MMC resin the Sj-p80 had a total purity of >99% with the primary band at ~80 kDa accounting for 87.8% of the total mainly due to a reduction in lower molecular weight degradation bands. Additionally, Sj-p80 at purity 4 had a significant reduction in *Ec*-HCPs (**Figures 1B, C**) and a five-fold reduction in endotoxin (**Table 1**). Sj-p80 at each purity level was analyzed by SDS-PAGE (**Figure 1B**, left panel) revealing that Sj-p80 purified predominantly as a single band at ~80 kDa with numerous smaller proteins in the 36–75 kDa region and very faint proteins in the ~148- to 250-kDa region. To deduce identity of these proteins, we performed Western analysis using a Sj-p80 specific mouse monoclonal antibody (SMab4) (**Figure 1B**, middle panel). We concluded that bands in the ~250-kDa region were likely comprised of Sj-p80 aggregates while the bands below 80 kDa were likely Sj-p80 degradation products.

To estimate the levels of residual of *E. coli* host cell proteins (HCPs), we performed an anti-*Ec*-HCP Western as shown in **Figure 1B** (right panel). All purity levels had very little HCP left, and any detected bands were distinct from the degradation products seen in the anti-Sj-p80 Western blot. For calculations of Sj-p80 purity in **Table 1**, we reported densitometry-based purity analyses based on two assumptions: 1) only considering the primary ~80 kDa band, or 2) including the ~80 kDa band plus the Mab-reactive degradation and aggregation bands. By this analysis, we determined that the purity of 80 kDa Sj-p80 band ranged from 60.7% to 87.8% but if all Mab-reactive bands were included in the purity analysis, we estimated that the purities of Sj-p80 antigen in the preparations to be up to 99%.

### Cost Analysis of Minimally Purified Sj-p80

Cost modeling studies were performed at the 10 L scale estimating costs of growth and expression, purification, personnel time, and reagents. We estimated the costs to produce the Sj-p80 for each purity level and divided this number by the protein yield in milligrams to estimate a cost per milligram. We estimated four bovine doses per milligram immunizing with 0.25 mg per dose. As shown in **Table 1**, at current scale and yields Sj-p80 Purity 1 cost approximately $5920 USD to produce an average of 508 mg for a cost per dose of $2.91 USD. By comparison, Sj-p80 at purity 4 protein cost of $17,520 USD to produce 224 mg resulting in a final cost of $19.55 USD per bovine dose. These comparisons were based on 3 independent purifications at the 10-L scale which resulted in average protein yields of 508 mg, 486, 448, and 224 mg for purities 1 to 4, respectively.

### Sj-p80-Specific Antibody Titers Following Vaccination

**Figure 2:**
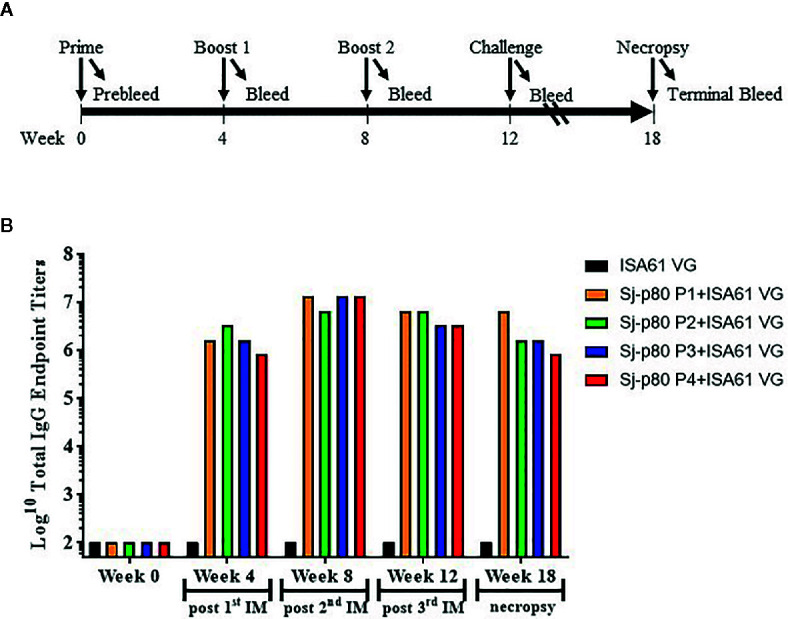
Vaccination strategy and kinetics of IgG production in mice. **(A)** Three injections (prime and 2 boosts) were performed 4 weeks apart, and mice were challenged with *S. japonicum* cercaria 4 weeks following the second boost. Necropsies were performed 6 weeks following challenge. **(B)** The kinetics of Sj-p80-specific total IgG following vaccination with each of 4 purities of Sj-p80 antigen in the first mouse experiment. Titers of anti-Sj-p80 total IgG were determined by ELISA following pooling of sera from all mice in the group. ELISA assays were conducted in triplicate and the values represents mean titer ± standard deviation.

The production of Sj-p80-specific antibodies following the vaccination schedule was compared between control and experimental animals. As shown in **Figure 2A**, mice were immunized three times at 4-week intervals (day 0, week 4 and week 8) and then challenged 4 weeks later. As shown in **Figure 2B**, all four Sj-p80 purities resulted in similar levels of total IgG at each time point with week 4 titers ranging from 819,200 to 3,276,800 and peak end-point titers observed after the first boost (week 8) ranging from 6,553,600 to 13,107,200. Titers at week 12 and after challenge on week 18 remained consistent with slight, albeit not statistically significant, variation between the various purities.

### Reduction in Parasitological Burden Following Sj-p80 Vaccination

**Figure 3:**
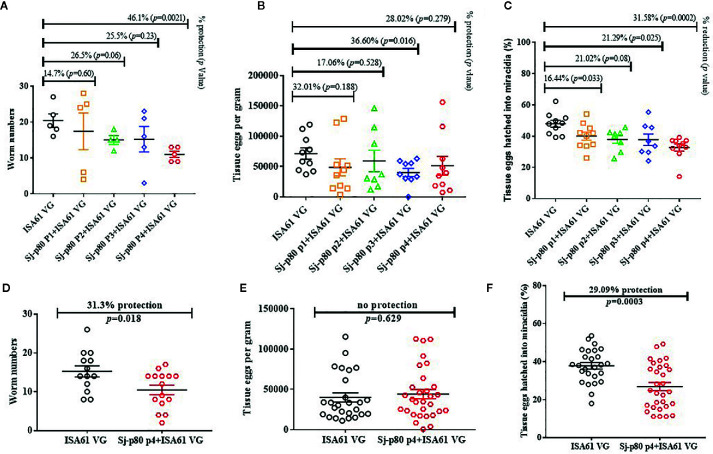
Sj-p80-based vaccine mediated protection in mice. **(A)** Adult worm numbers recovered per mouse in the Montanide™ ISA61 VG control group (ISA61 VG) and Sj-p80 + Montanide™ ISA61 VG experimental groups in Trial 1. **(B)** Egg load per gram of tissue (liver and intestine) and **(C)** percentage tissue eggs per gram hatched into miracidia in Trial 1. **(D)** Adult worm numbers recovered per mouse in the Montanide™ ISA61 VG control group (ISA61 VG) and Sj-p80 P4 + Montanide™ ISA61 VG experimental groups in Trial 2. **(E)** Egg load per gram of tissue (liver and intestine) and **(F)** percentage tissue eggs per gram hatched into miracidia in Trial 2. Worm burden was determined 6 weeks following *Schistosoma japonicum* cercarial challenge. Sj-p80 P1, P2, P3, and P4 represents purity levels 1, 2, 3 and 4, respectively. Each mouse was challenged with 40 *S. japonicum* cercariae. *p* ≤ 0.05 was considered significant. In panels **(B, C, E, F)** the liver and intestine egg values are shown as separate data points on the graphs.

In the first mouse experiment, four groups of mice (*n*=5) received Sj-p80 at different purities along with the adjuvant Montanide™ ISA61 VG. Mice were challenged with *S. japonicum* cercariae and infection was allowed to progress for six weeks. Results showed that all purities of Sj-p80 conferred moderate to significant protection against *S. japonicum* infection in mice (**Figure 3**). One mouse immunized with Sj-p80 purity 2 died of unknown causes early in the study. Sj-p80 vaccine at the highest level of purity demonstrated the highest prophylactic effect in immunized mice as evidenced by a significant reduction of adult worm numbers (46.1%, *p*=0.0021) when compared to the control group (**Figure 3A**). Each of the other three less pure vaccines resulted in moderate reduction in adult worm numbers, however not significant (**Figure 3A**). Mice immunized with Sj-p80 purity 2 or purity 4 showed statistically significant reductions in female worms of 31.55% (*p* = 0.035) and 37.7% (*p* = 0.022), respectively. Mice receiving Sj-p80 purity 3 showed a reduction of 21.43% which was not statistically significant (*p* = 0.36), and mice immunized with Sj-p80 purity 1 showed a very slight and not statistically significant reduction (2.4%, *p* = 0.94). We also observed a moderate reduction in gross tissue (liver and intestine) eggs per gram in experimental groups when compared to the control (**Figure 3B**). Specifically, we observed a significant reduction in liver egg burden (51.4%, *p* = 0.02) in mice immunized with Sj-p80 purity 4 with moderate reduction of 32.4% (*p* = 0.15), 44.3% (*p* = 0.03) and 23.4% (*p* = 0.23) in animals that received Sj-p80 purities 1, 2, and 3 respectively (**Supplementary Figure 1A**). Similarly, there was also a reduction in intestine egg load in mice immunized with Sj-p80 purity 1 (31.8%, *p* = 0.34), purity 2 (1.9%, *p* = 0.95), purity 3 (55.1%, *p* = 0.02) and purity 4 (15.0%, *p* = 0.64) (**Supplementary Figure 1B**). Interestingly, we found a significant reduction in the ability of recovered gross tissue eggs (liver and intestine) to hatch into miracidia with 3 of the 4 purity levels (**Figure 3C**). Hatching rates of eggs recovered from mice immunized with Sj-p80 purity 4 showed the greatest reduction at 31.6% reduction (*p* = 0.0002). Sj-p80 at purities 1, 2, and 3 also showed reductions of 16.4% (*p* = 0.033), 21.0% (*p* = 0.08), and 21.3% (*p* = 0.025), respectively. Specific liver and intestine eggs hatching rates from mice immunized with Sj-p80 vaccine at purities 1, 2, 3, and 4, respectively, are presented in **Supplementary Figures 1C, D**.

To confirm these results, we performed a second challenge study comparing only purity level 4 with the adjuvant only group and increased the group sizes to 15 animals to allow for better statistical analyses. During this experiment, two mice in the control group died of unknown causes. The results were a 31.3% reduction (*p* = 0.018) in total worm numbers (**Figure 3D**) including a significant reduction of 23.0% in female worms (*p* = 0.013). Despite the reduction in female worms, we did not observe any reduction in gross tissue eggs per gram (**Figure 3E**). However, consistent with results obtained in the first experiment, we again observed a significant reduction of 29.1% (*p*=0.0003) in ability of the eggs to hatch into miracidia.

### Determination of Antibody Subtypes

**Figure 4:**
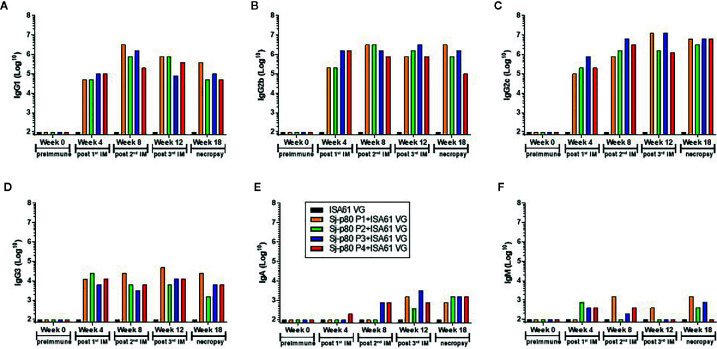
Titer of anti-Sj-p80 antibody subtypes in immunized mice. Antibody titers for **(A)** IgG1, **(B)** IgG2b, **(C)** IgG2c, **(D)** IgG3, **(E)** IgA and **(F)** IgM at weeks 0, 4, 8, 12 and 18 were measured by ELISA using isotype specific antibodies following pooling of sera from all mice in the group. Purities of Sj-p80 antigen are indicated as follows: purity 1 (orange bars), purity 2 (green bars), purity 3 (blue bars), purity 4 (red bars). All assays were conducted in replicates and the values represents mean ± standard error of mean.

Significant antibody responses were also observed for all Sj-80-specific IgG subtypes including IgG1 (maximum end-point titer 3,276,800) (**Figure 4A**), IgG2b (maximum end-point titer 3,276,800) (**Figure 4B**), IgG2c (maximum end-point titer 6,553,600 - 13,107,200) (**Figure 4C**), and IgG3 (maximum end-point titer, 12,800–51,200) (**Figure 4D**). There was only a mild (2–3 log) increase in the production of IgA and almost no induction of IgM at the timepoints tested (with maximum pre-sacrifice titers of 3,200 and 1,600, respectively) (**Figures 4E, F**). In addition, we did not observe any significant differences in the levels of any subtype that correlated to the 4 purities.

**Figure 5:**
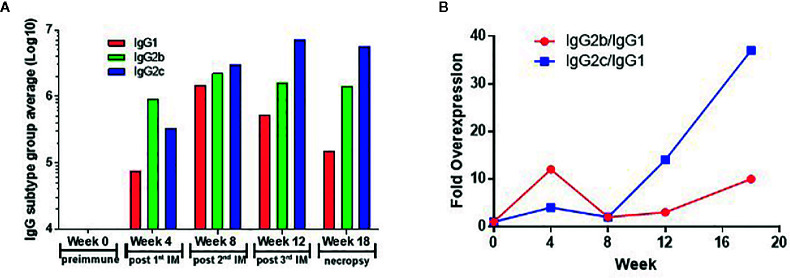
Th1 skewing induced by vaccination with Sj-p80 plus Montanide™ ISA61 VG. Panel **(A)** shows the end-point titers of IgG1 (red bars), IgG2b (green bars), and IgG2c (blue bars) following averaging of individual end-points titers of all 4 purities. Panel **(B)** shows the mathematical ratio of the IgG2b titers divided by the IgG1 titers (red line) or the IgG2c titers divided by the IgG1 titers (blue line).

To identify whether the Sj-p80 vaccine was inducing a Th1 or Th2 biased response, we analyzed the antibody response by subclass. **Figure 5** shows the expression of IgG1, IgG2b, and IgG2c isotypes following averaging of the individual responses from all 4 purities. The Sj-p80 vaccine formulation used clearly promotes a Th1-biased response as indicated by the slow increase of the Th2 marker IgG1 to a maximum at week 8 followed by a sharp decline at weeks 12 and 18 (**Figure 5A**). By contrast, both Th1 associated isotypes IgG2b and IgG2c were induced more quickly and their overall levels continued to increase throughout the study until sacrifice. To highlight the overexpression of IgG2b relative to IgG1, we divided the end-point titers of IgG2c by that of IgG1 (**Figure 5B**, blue line). The result showed an approximately 15-fold abundance of IgG2c at 4 weeks which dropped to ~4-fold at week 8. However, after the second boost a ~16-fold abundance was observed at week 12 which continued to increase following challenge to a final ~46-fold abundance. A similar - albeit less marked - trend was observed when comparing the level of IgG2b to IgG1 (**Figure 5B**, red line) in which a ~10-fold abundance was seen at week 4 followed by similar titers at week 8. Again, after the second boost and continuing through challenge, the overexpression of IgG2b relative to IgG1 was 4-fold at week 12 and 10-fold at week 18. These data clearly suggest that a Th1-biased response is being elicited by this Sj-p80 vaccine.

### Cytokine Expression Profiles Following Sj-p80 Vaccination

**Figure 6:**
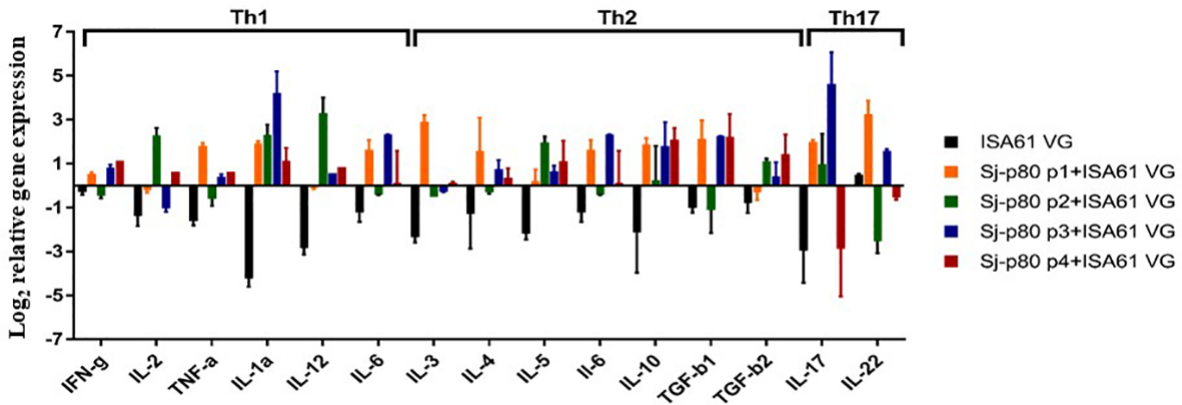
Cytokine mRNA expression profiles in stimulated splenocytes of mice vaccinated with Sj-p80. The expression profile of Th1, Th2 and Th17 type cytokines was determined by qRT-PCR. The relative mRNA expression was calculated after normalization to the expression of glyceraldehyde 3-phosphate dehydrogenase. Total RNA used was obtained from pooled splenocytes from mice belonging to each group. All experiments were conducted in triplicate.

Consistent with the antibody results, quantitative RT-PCR analysis of a panel of Th1/Th2/Th17 cytokines tested showed that there was an increase in the expression of some Th1-biased cytokines in mice immunized with Sj-p80 + Montanide™ ISA61 VG compared to the control animals (**Figure 6**). Specifically, we observed moderate upregulation in the expression of IFN-*γ*, IL-2, TNF-α, IL-1a, IL-12, and IL-6 in Sj-p80-stimulated splenocytes obtained from experimental mice when compared to their control counterparts in which these cytokines were downregulated. Of the Th1-biased cytokines, only IL-1a and IL-12 were upregulated when stimulated with all 4 Sj-p80 purities, and IL-2 was actually downregulated with 2 of the purities and upregulated with the other 2 (**Figure 6**). Similarly, analysis of the Th2 and Th17-dependent cytokine expression also showed an overall significant increase in all of the cytokines tested including IL-4, IL-5, IL-10, TGF-β, IL-17, and IL-22 with only IL-3 expression remaining effectively unchanged.

## Discussion

Asiatic schistosomiasis caused by *S. japonicum* is an important disease affecting both humans and animals. Infections in bovines such as water buffalo and yellow cattle are especially devastating given their importance in China and Southeast Asia for farming as well as a direct source of milk and meat (31). Infected bovines are believed to be the most significant source of infective eggs through excretion of feces resulting in perpetuation of the complex lifecycle. The inadequacies of current control programs highlight the need for an effective vaccine (18, 51). An effective transmission-blocking veterinary vaccine focusing on bovines would likely disrupt this cycle likely leading to reduced human infections (12).

One vaccine under development for Asiatic schistosomiasis is Paramyosin (rSj97) (29, 31, 52) [reviewed in You et al. (12)]. This vaccine has shown promise in early field trials in sheep, pigs, and water buffaloes [reviewed in (12)] and more recent trials in 2016 with rSj97 paired with Montanide ISA206 showed promising results (29, 31). A second promising candidate in water buffalo is the triose-phosphate isomerase (TPI) antigen fused to heat shock protein 70 (Hsp70) which in water buffalo reduced worm burden by 51.2%, reduced liver eggs by 61.5%, and reduced egg hatching by 52.1% (27). While promising, both vaccines have been slow to progress beyond preliminary studies for unknown reasons. Some of the roadblocks that hamper development may be high production costs, high dose requirement, requirement for multiple boosts, and limitations in funding for developing a zoonotic vaccines.

The role of antibodies in immune protection against schistosomiasis has also been well documented with studies utilizing non- or semi-permissive hosts demonstrating that resistance is almost entirely antibody-dependent (53–55). In our experimental groups, immunization of mice with Sj-p80 + Montanide™ ISA61 VG induced robust humoral immune responses including IgG1 [linked to T-helper cell 2 (Th2) phenotype] and much more pronounced IgG2 subclass antibody production indicative of a robust Th1 response (56, 57). We also found an induction of Sj-p80-specific IgA antibodies in the serum of immunized animals. These antibodies may play a role in mucosal immunity to the disease given that schistosome eggs are in constant contact with the gut-associated mucosal system.

The pathology of schistosomiasis and its severity is linked to the host immune responses to antigens secreted by viable schistosome eggs and the number of eggs trapped within the host tissues, particularly the liver (58, 59). In our study, mice immunized with Sj-p80 had a substantial reduction in overall worm numbers and preferential killing of female worms. However, we did not observe any reduction in tissue eggs in either experiment. Of particular interest is the observed reduction in egg viability/hatching rates in eggs obtained from the tissues (liver and intestines) of vaccinated animals. Specifically, eggs obtained from mice immunized with Sj-p80 had a 32% reduction in hatching rate supporting the potential transmission-blocking effects of the vaccine. In addition, since the severity of schistosomiasis is also associated with the number of viable eggs trapped in host tissues, the Sj-p80 vaccine-mediated reduction in egg hatching capabilities affirms its anti-pathology efficacy (60, 61).

In addition to the critical roles played by host humoral immunity in schistosomiasis resistance, studies have shown that certain cellular immune responses, specifically Th1-type immunity, also play an integral role (62, 63). We observed increased expression of Th1-type cytokines (*IFN-*γ*, IL-2, TNF-α*, and *IL-12*) from spleen cells isolated from experimental group mice when stimulated *ex vivo* with Sj-p80 antigen. We also observed an upregulation of *IL-4, IL-5, IL-6, IL-10*, and *TGF-β* genes in splenocytes from vaccinated mice indicating a mixed Th1/Th2 response. Cumulatively, we conclude that Sj-p80 vaccine formulated in Montanide™ ISA61 VG induced both Th1 and Th2-type immune responses associated with protection in immunized mice.

Cost analysis of minimally purified Sj-p80 paired with a low-cost adjuvant such as Montanide™ ISA61 VG resulted in a vaccine cost between $3.00 and $20.00 per bovine dose when produced at small scale (10 L). We envision that scaling up to 1,000 L during commercial manufacturing will reduce costs up to 10-fold resulting in a final embodiment of the vaccine which can be produced more cheaply. From the cumulative results here, we envision testing purities 2, 3, and 4 in our preliminary bovine experiments. The final actual vaccine cost will be dependent on several factors including the final chosen purity, scale of manufacture, and number of doses required to achieve protection.

We report here, for the first time, a Sj-p80-based vaccine that protects against *S. japonicum* parasite challenge in mice and can be produced at a viable deployment cost. The ultimate market we intend to target is bovines, particularly water buffalo, in regions of the world where Asiatic schistosomiasis caused by *S. japonicum* is most prevalent. The preliminary studies here therefore have set the stage for scaling and testing in bovines in endemic settings in preparation for deployment of a new veterinary vaccine for *S. japonicum*.

## Data Availability Statement

The raw data supporting the conclusions of this article will be made available by the authors, without undue reservation.

## Ethics Statement

The animal study was reviewed and approved by TTUHSC IACUC Protocol Number 20010202.

## Author Contributions

SG, DC, and AS conceived the study while SG and AM designed the experiments. CT, JD, and SG performed recombinant production of Sj-p80. AM, WZ, SK, and SS performed mice experiments. AM, SK, and WZ carried out all parasitological assays. AM, MR, RK, and CP carried out all ELISA and AM and MR carried out qRT-PCR experiments. The manuscript was written by AM and SG. All authors contributed to the article and approved the submitted version.

## Funding

This work was supported by SBIR grant (R43AI142908) to SG (PI) and AS (MPI).

## Conflict of Interest

SG, CT, JD, and DC were all employed by the company PAI Life Sciences, Inc.

The remaining authors declare that the research was conducted in the absence of any commercial or financial relationships that could be construed as a potential conflict of interest.

## Supplementary Material

Supplementary Figure 1
*Schistosoma japonicum* egg burden and percent egg from tissues hatched into miracidia. **(A)** Egg load per gram of liver per mouse in the Montanide™ ISA61 VG control group (ISA61 VG) and Sj-p80 + Montanide™ ISA61 VG experimental groups in Trial 1. **(B)** Egg load per gram of intestine per mouse in the Montanide™ ISA61 VG control group (ISA61 VG) and Sj-p80 + Montanide™ ISA61 VG experimental groups in Trial 1. **(C)** Percent eggs from liver hatched into miracidia per mouse in the Montanide™ ISA61 VG control group (ISA61 VG) and Sj-p80 + Montanide™ ISA61 VG experimental groups in Trial 1. **(D)** Percent eggs from intestine hatched into miracidia per mouse in the Montanide™ ISA61 VG control group (ISA61 VG) and Sj-p80 + Montanide™ ISA61 VG experimental groups in Trial 1. Egg burden and hatching rates were determined 6 weeks following *Schistosoma japonicum* cercarial challenge. Sj-p80 P1, P2, P3, and P4 represents purity levels 1, 2, 3 and 4, respectively. Each mouse was challenged with 40 *S. japonicum* cercariae. *p* ≤ 0.05 was considered significant. Egg per gram **(A, B)** and egg hatching rates **(C, D)** for each mouse are shown as separate data points on the graphs.
