# Supplementary figures and images for: Process Development of Sj-p80: A Low-Cost Transmission-Blocking Veterinary Vaccine for Asiatic Schistosomiasis
Source: Front Immunol. 2021 Feb 23;11:578715. doi: 10.3389/fimmu.2020.578715 (PMC7959798; doi:10.3389/fimmu.2020.578715)

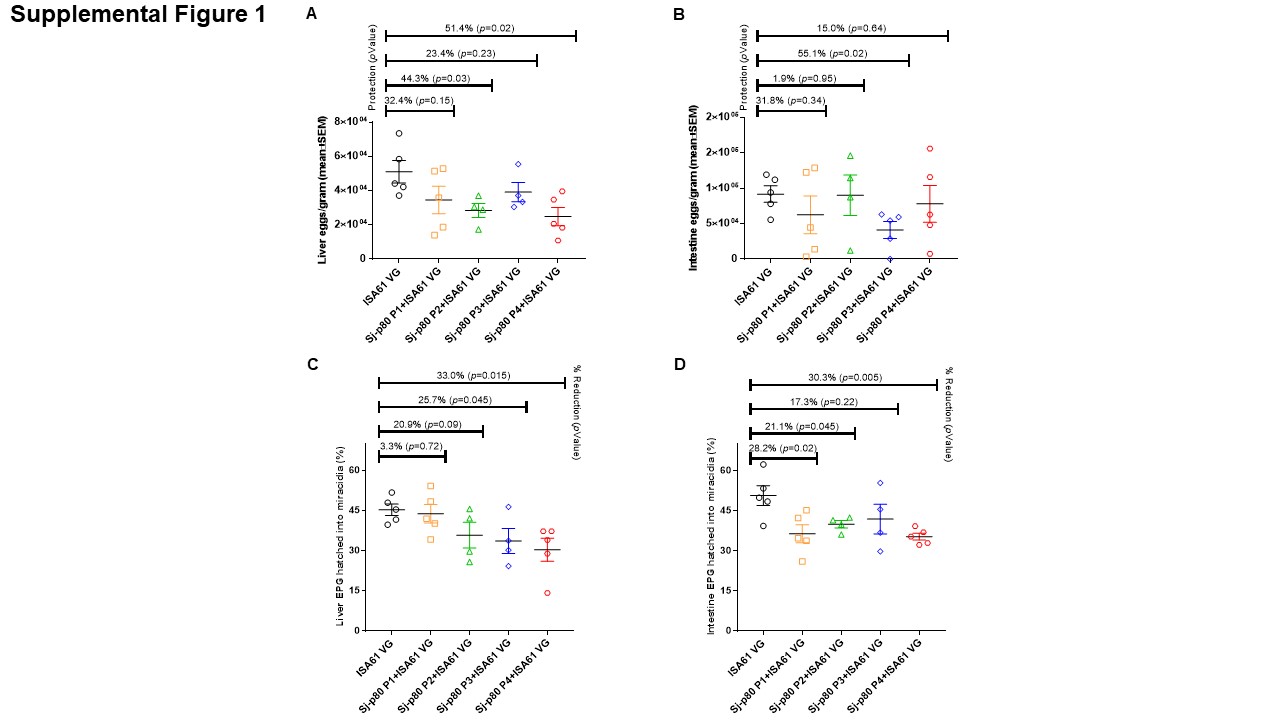

Supplement: Supplementary Figure 1 — Schistosoma japonicum egg burden and percent egg from tissues hatched into miracidia. (A) Egg load per gram of liver per mouse in the Montanide™ ISA61 VG control group (ISA61 VG) and Sj-p80 + Montanide™ ISA61 VG experimental groups in Trial 1. (B) Egg load per gram of intestine per mouse in the Montanide™ ISA61 VG control group (ISA61 VG) and Sj-p80 + Montanide™ ISA61 VG experimental groups in Trial 1. (C) Percent eggs from liver hatched into miracidia per mouse in the Montanide™ ISA61 VG control group (ISA61 VG) and Sj-p80 + Montanide™ ISA61 VG experimental groups in Trial 1. (D) Percent eggs from intestine hatched into miracidia per mouse in the Montanide™ ISA61 VG control group (ISA61 VG) and Sj-p80 + Montanide™ ISA61 VG experimental groups in Trial 1. Egg burden and hatching rates were determined 6 weeks following Schistosoma japonicum cercarial challenge. Sj-p80 P1, P2, P3, and P4 represents purity levels 1, 2, 3 and 4, respectively. Each mouse was challenged with 40 S. japonicum cercariae. p ≤ 0.05 was considered significant. Egg per gram (A, B) and egg hatching rates (C, D) for each mouse are shown as separate data points on the graphs. [file Image_1.jpeg]
