# Supplementary material for: Process Development of Sj-p80: A Low-Cost Transmission-Blocking Veterinary Vaccine for Asiatic Schistosomiasis
Source: Front Immunol. 2021 Feb 23;11:578715. doi: 10.3389/fimmu.2020.578715 (PMC7959798; doi:10.3389/fimmu.2020.578715)
Supplement: Supplementary file 2 [file Table_1.pdf]

Supplementary Table 1. Cytokine primer sequences.		
**Gene**		**Primer sequence**
GAPDH	Sense	5′- AAC AGC AAC TCC CAC TCT TC -3′
	Antisense	5′- CCT GTT GCT GTA GCC GTA TT -3′
IFN-γ	Sense	5′- ACT GGC AAA AGG ATG GTG AC -3′
	Antisense	5′- GAC CTG TGG GTT GTT GAC CT -3′
TNF-α	Sense	5′- TCT TCT CAT TCC TGC TTG TGG -3′
	Antisense	5′- GGT CTG GGC CAT AGA ACT GA -3′
IL-2	Sense	5′- CCC AGG ATG CTC ACC TTC AA -3′
	Antisense	5′- CCG CAG AGG TCC AAG TTC AT -3′
IL-12	Sense	5′- CTG TGC CTT GGT AGC ATC TAT G -3′
	Antisense	5′- GCA GAG TCT CGC CAT TAT GAT TC -3′
IL-1α	Sense	5′- TGA AGT TGA CGG ACC CCA AA -3′
	Antisense	5′- TGA TGT GCT GCT GCG AGA TT -3′
IL-6	Sense	5′- ATG GAT GCT ACC AAA CTG GAT -3′
	Antisense	5′- TGA AGG ACT CTG GCT TTG TCT -3′
IL-4	Sense	5′- GAA GAA CAC CAC AGA GAG TGA GC -3′
	Antisense	5′- CTT TCA GTG ATG TGG ACT TGG AC -3′
IL-3	Sense	5′- ACA ATG GTT CTT GCC AGC TC -3′
	Antisense	5′- GCT GCA ATT CAA CGT TCT GG -3′
IL-5	Sense	5′- CTC TGT TGA CAA GCA ATG AGA CG -3′
	Antisense	5′- TCT TCA GTA TGT CTA GCC CCT G -3′
TGF-β1	Sense	5′- TGC GCT TGC AGA GAT TAA AA -3′
	Antisense	5′- GCT GAA TCG AAA GCC CTG TA -3′
TGF-β2	Sense	5′- AGC TTC ATG GAC GCC ATA TG -3′
	Antisense	5′- TCA ACA TTT CAC CGG CTT GC -3′
IL-17	Sense	5′- GCT CCA GAA GGC CCT CAG A -3′
	Antisense	5′- AGC TTT CCC TCC GCA TTG A -3′
IL-22	Sense	5′- GGC CAG CCT TGC AGA TAA CA -3′
	Antisense	5′- GCT GAT GTG AGA GGA GCT GA -3′
